# Acute effect of lower‐limb peristaltic pulse external pneumatic compression on segmental arterial stiffness in healthy young adults

**DOI:** 10.14814/phy2.70380

**Published:** 2025-05-19

**Authors:** Daisuke Kume, Masato Nishiwaki, Tomomi Monri

**Affiliations:** ^1^ Faculty of Information Science and Technology Osaka Institute of Technology Osaka Japan; ^2^ Faculty of Engineering Osaka Institute of Technology Osaka Japan; ^3^ Comprehensive Education Center Kawasaki University of Medical Welfare Okayama Japan

**Keywords:** cardio‐ankle vascular index, mood, pulse wave velocity, sympathetic nerve activity

## Abstract

Although an acute bout of peristaltic pulse external pneumatic compression (EPC) of the lower extremities can improve vascular function, its effect on arterial stiffness remains unclear. This study aimed to investigate the acute effect of lower‐limb peristaltic pulse EPC on segmental arterial stiffness. Nineteen healthy young adults (12 males and 7 females) (age: 20 ± 1 years) were allocated to undergo two experimental trials using a randomized crossover design. EPC treatment delivered at 70–80 mmHg (EPC trial) or non‐compressed sham treatment (sham trial) was performed for 30 min. At baseline (Pre) and after the 30‐min treatment (Post), arterial stiffness indices, including the heart‐brachial pulse wave velocity (PWV) (hbPWV), brachial‐ankle PWV (baPWV), heart‐ankle PWV (haPWV), and the cardio‐ankle vascular index (CAVI) were measured simultaneously. All parameters in the EPC trial exhibited a significant decrease (*p* < 0.05) at Post compared to Pre, including hbPWV, baPWV, haPWV, and CAVI. However, no such changes were observed in the sham trial. The study findings demonstrate that a single 30‐min bout of lower‐limb peristaltic pulse EPC can decrease arterial stiffness in healthy young adults. The observed EPC‐induced decrease in arterial stiffness in various segments suggests that the beneficial vascular effect could be elicited systemically.

## 1 INTRODUCTION

Increased arterial stiffness has been identified as an independent risk factor for future cardiovascular disease (Laurent & Boutouyrie, 2007), and pulse wave velocity (PWV) is a standard measure of arterial stiffness (Townsend et al., 2015). In addition to aortic PWV (Vlachopoulos et al., 2010), PWVs of other arterial segments have recently been shown to be associated with cardiovascular disease risk (Stone et al., 2022, 2023; Sugawara et al., 2024). Arterial stiffness increases with advancing age even in healthy individuals (Nishiwaki et al., 2014; Tanaka et al., 2009), making the prevention of vascular stiffening an important endeavor for the maintenance of vascular health, irrespective of age. Evidence indicates that regular aerobic exercise, such as cycling or running, is effective in reducing arterial stiffness (Seals et al., 2019; Tanaka, 2019). However, people who have partial paralysis or arthritis are unable to perform these kinds of lower‐limb exercises. Additionally, even in healthy individuals, perceived barriers to exercise can hinder regular implementation (Peng et al., 2023; Rhodes et al., 1999). As such, establishing simple and effective passive approaches for reducing arterial stiffness is an attractive proposition.

Lower‐limb compression (e.g., enhanced external counterpulsation) has traditionally been used as a therapeutic strategy in vascular medicine (Braith et al., 2012). Recently available commercial external pneumatic compression (EPC) devices utilize whole‐leg sleeves that operate by inflating and deflating a series of zones, which can provide peristaltic pulse compression (Haun et al., 2017). The use of EPC devices is putatively analogous to lower‐extremity massage, which has a beneficial vascular effect (Franklin et al., 2014). Martin et al. (2015) previously demonstrated that a single, 1‐h bout of EPC treatment yields an acute improvement in endothelial function, as evaluated using flow‐mediated dilation (FMD). Notably, this improvement was elicited in both the popliteal (treated leg) and brachial (untreated arm) arteries (Martin et al., 2015), implying the presence of a systemic effect. These observations were supported by a subsequent study that found that 30 min of unilateral EPC increased popliteal FMD in the treated and untreated legs (Martin et al., 2018). Given that acute changes in endothelial function could affect arterial stiffness (Stoner et al., 2020), it is reasonable to speculate that acute administration of EPC would result in the amelioration of arterial stiffness; however, this aspect remains to be investigated.

We previously assessed segmental arterial stiffness responses to acute physiological stimuli in our prior studies (Ikebe et al., 2022; Kume et al., 2020, 2021, 2022a, 2022b; Oda et al., 2022). Therefore, the present study aimed to examine the acute effect of lower‐limb peristaltic pulse EPC on segmental arterial stiffness. To attain this objective, we assessed the responses in arterial stiffness in various segments to 30 min of EPC treatment, and the values were compared with those of non‐compressed sham treatment. Since it has been suggested that changes in sympathetic nerve activity or psychological status could affect arterial stiffness (Nardone et al., 2020; Vlachopoulos et al., 2009), relevant physiological and psychological parameters were also evaluated in this setting. We hypothesized that an acute bout of EPC would systemically ameliorate arterial stiffness.

## 2 METHODS

### 2.1 Ethical approval

This study was approved by the Human Ethics Committee at the Osaka Institute of Technology (approval number: 2024‐19) and conducted in accordance with the tenets of the Declaration of Helsinki. All participants provided written informed consent, after receiving a complete explanation about the study's purpose, experimental procedure, and associated risks.

### 2.2 Participants

This study employed a randomized crossover trial design. The sample size required for this study was calculated using G*Power version 3.1 (Heinrich Heine University, Düsseldorf, Germany). The effect size (f) was set to 0.25 (medium) (Cohen, 1992) using a within–between interaction of the two‐way repeated‐measures analysis of variance (ANOVA) (number of trials = 2, number of measurements = 2). The α‐ and β‐levels were set to 0.05 and 0.2 (80% power), respectively. Based on this calculation, the minimum number of participants required was determined to be 17.

Nineteen healthy young adults (12 males and 7 females) participated in this study. Their physical characteristics are shown in Table 1. All participants were nonsmokers and were not presently taking any medication or supplements. They were not obese (body mass index <30 kg/m^2^) or hypertensive (systolic blood pressure < 140 mmHg or diastolic blood pressure < 90 mmHg).

**TABLE 1:** Physical characteristics of study participants.

Number of participants	Total (*n* = 19)	Males (*n* = 12)	Females (*n* = 7)
Age (years)	21 ± 1	21 ± 1	21 ± 1
Height (cm)	167 ± 7	170 ± 5	161 ± 6
Body weight (kg)	58 ± 7	61 ± 5	53 ± 6
Body mass index (kg/m^2^)	20.9 ± 1.7	21.2 ± 1.6	20.5 ± 1.8

*Note*: Data are expressed as mean ± standard deviation.

### 2.3 Experimental procedures

The participants visited the laboratory three times throughout the experimental period. During the first visit, participants were familiarized with the experimental apparatus. During the second and third visits (i.e., experimental visits), the participants underwent the EPC and sham trials. The order of the experimental visits was randomly allocated, and the interval between consecutive examinations was at least 7 days. To avoid any potential diurnal effects, experiments were conducted at the same time of the day for each participant. The participants were asked to refrain from performing strenuous exercise, consuming alcohol (≥24 h) and caffeine (≥12 h), and eating (≥3 h) before the trials; compliance with these requirements was confirmed using a checklist questionnaire and face‐to‐face interview. All experiments were conducted in a quiet, air‐conditioned room (24–26°C).

**FIGURE 1:**
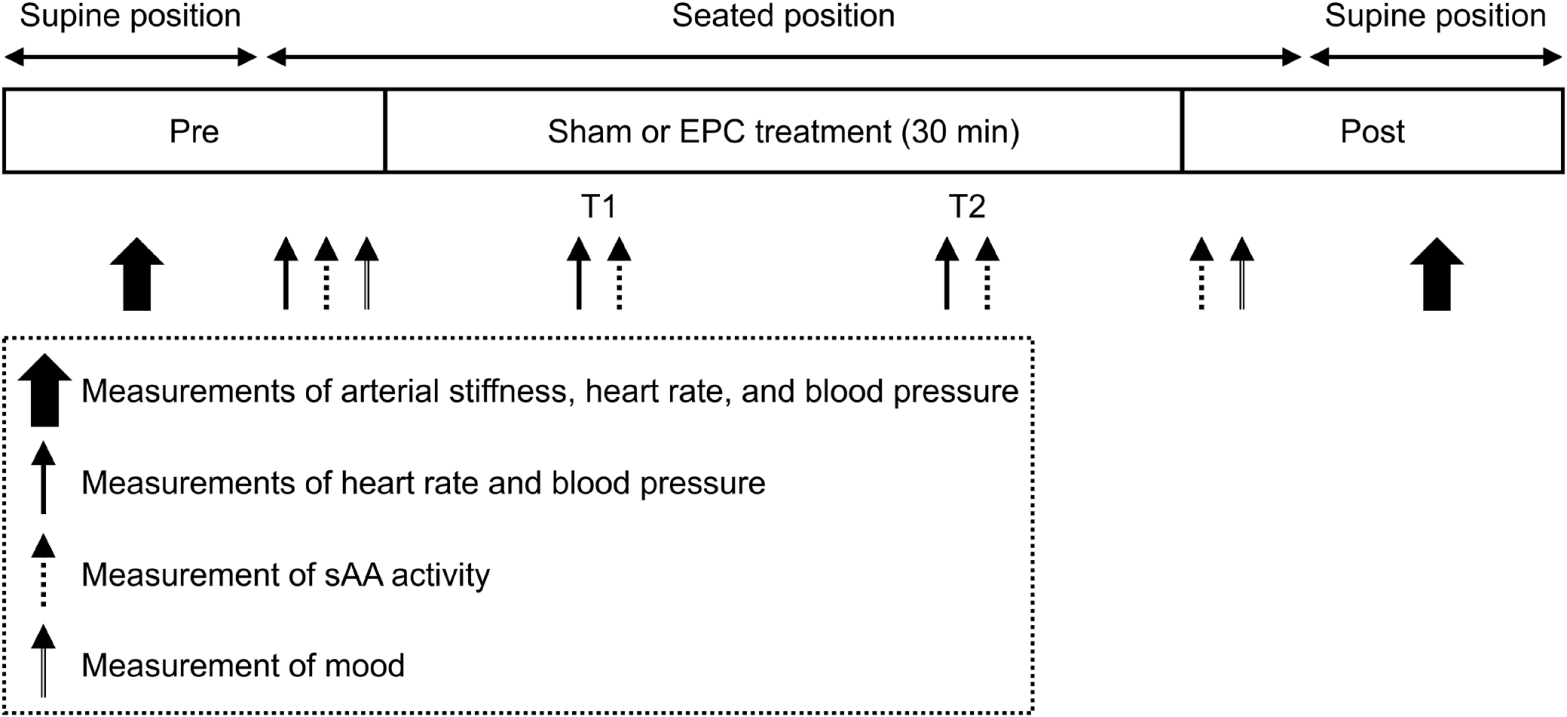
Experimental protocol. T1 and T2, 10–12 and 20–22 min following treatment initiation, respectively. EPC, external pneumatic compression; sAA, salivary alpha‐amylase.

A schematic representation of the experimental protocol of the present study is presented in Figure 1. The participants' pretrial baseline measures of arterial stiffness and hemodynamic variables were measured in the supine position after a 15‐min rest period. Thereafter, the participants sat in a comfortable recliner chair with their legs extended, and a peristaltic pulse dynamic EPC device (NormaTec; Hyperice, Irvine, CA, USA) was attached to their lower extremities. The device consists of two separate leg sleeves that contain five circumferential inflatable chambers (arranged linearly along the limb) encompassing the leg from the feet to the hip/groin. The leg sleeves are connected to an automated pneumatic pump at which target inflation pressures for each zone, and the duty cycle can be controlled. The participants rested in that state for 5 min, followed by the assessment of hemodynamic variables, salivary alpha‐amylase (sAA) activity, and mood (Pre). Next, in the EPC trial, the participants underwent EPC treatment for 30 min. The target inflation pressures were set at 70–80 mmHg for each chamber (Martin et al., 2016), where the duty cycle comprised 30 s of compression in each zone, followed by a 30‐s rest period during which all zones were deflated (Haun et al., 2017; Martin et al., 2016, 2018). An illustration of the compression pattern delivered by the EPC device has been presented elsewhere (Haun et al., 2017). The EPC protocol used in this study has been previously confirmed to acutely improve vascular function (Martin et al., 2018). In contrast, the participants in the sham trial underwent a 30‐min application of the EPC leg sleeves, which were connected to the pneumatic pump, but without actual compression; this was employed to control for any thermogenic effect of wearing the leg sleeves (Martin et al., 2015). At approximately 10–12 min (T1) and 20–22 min (T2) following the initiation of treatment, hemodynamic variables and sAA levels were measured. Immediately after the cessation of treatment, the sAA levels and mood were measured (Post). Lastly, post‐trial measurements of arterial stiffness and hemodynamic variables were performed with the participants in the supine position.

### 2.4 Measurements

#### 2.4.1 Arterial stiffness and hemodynamic variables (in the supine position)

Arterial stiffness measures, heart rate, systolic and diastolic blood pressure, and mean arterial pressure were measured using a vascular screening system (VaSera VS‐1500AN; Fukuda Denshi, Tokyo, Japan). The VaSera system has been used in our and other previous studies (Ikebe et al., 2022; Kume et al., 2020, 2021, 2022a, 2022b; Nishiwaki et al., 2014; Oda et al., 2022; Sugawara et al., 2018). For the measurements, blood pressure cuffs were wrapped around both the upper arms and ankles. Phonocardiography and electrocardiography electrodes were placed on the pectoral region and on both wrists, respectively. Electrocardiography, heart sounds, and arterial pressure waveforms at the brachial and posterior‐tibial arteries were simultaneously recorded. According to the methods used in our previous studies (Kume et al., 2020, 2021, 2022a, 2022b), we measured the heart‐brachial PWV (hbPWV), brachial‐ankle PWV (baPWV), and heart‐ankle PWV (haPWV). hbPWV, baPWV, and haPWV were calculated from each arterial path length along with the time intervals between the second heart sound and the dicrotic notch on the brachial arterial pressure waveform; the time between the foot of the brachial arterial pressure waveform and the foot of the posterior‐tibial arterial pressure waveform; and the sum of these time intervals. Furthermore, the cardio‐ankle vascular index (CAVI) was calculated automatically. hbPWV reflects stiffness from the heart to the brachial artery (Sugawara et al., 2018, 2019). baPWV reflects the stiffness of the abdominal aorta and leg arteries (Stone et al., 2023; Sugawara et al., 2018). haPWV reflects the stiffness from the aorta to the ankle, and CAVI is a metric of haPWV adjusted by blood pressure (Hayashi et al., 2015). In our laboratory, the day‐to‐day coefficients of variation for hbPWV, baPWV, haPWV, and CAVI measurements on two separate days (i.e., reproducibility) were 2.3 ± 1.6%, 1.4 ± 1.0%, 1.8 ± 1.3%, and 2.2 ± 1.7%, respectively.

#### 2.4.2 Hemodynamic variables, sAA activity, and mood (in the seated position)

Blood pressure measures were obtained from the participant's left upper arm using an automated sphygmomanometer (Tango M2; SunTech Medical Instruments, Morrisville, NC, USA). The heart rate was also assessed using electrocardiography (Tango M2). Pre‐measurements were conducted twice, with a 60‐s interval between readings, and the average was used for analysis. During treatment, measurements were taken once at each time point (i.e., T1 and T2).

Since sAA levels are thought to reflect the activity of the sympathetic nerve system (Nater & Rohleder, 2009), sAA activity was used in the present study as a surrogate marker of sympathetic nerve activity. The measurements were conducted using an sAA monitor (Nipro, Osaka, Japan), whose validity has been confirmed previously (Shetty et al., 2011). A test strip that integrates a collector pad and a reagent paper containing α‐2‐chloro‐4‐nitrophenyl‐galactopyranosylmaltoside was placed under the participant's tongue for 30 s, after which the test strip was inserted into the reader. sAA levels were determined by photometrically measuring 2‐chloro‐4‐nitrophenol produced from α‐2‐chloro‐4‐nitrophenyl‐galactopyranosylmaltoside by amylase activity (Shetty et al., 2011).

A single session of standardized massage of the legs has been reported to produce an improvement in mood states (Noto et al., 2010). Moreover, it has been suggested that a positive psychological stimulus could have a beneficial impact on arterial stiffness (Vlachopoulos et al., 2009). Therefore, we assessed mood as follows: the participants rated their perceived levels of tension, anxiety, fatigue, and relaxation at that moment along a 0–10 Likert scale (Kume et al., 2022a; Sullivan et al., 2010).

### 2.5 Statistics

Data are expressed as mean ± standard deviation. The two‐way (time × trial) ANOVA with Bonferroni‐corrected post hoc testing was performed for all dependent variables in both trials. Statistical significance was set at a *p* value <0.05. The statistical analyses were conducted using SPSS version 28.0 (IBM SPSS Japan, Tokyo, Japan). Regarding the arterial stiffness measures, the effect size (dz) for Pre–Post changes was calculated using G*Power version 3.1.

## 3 RESULTS

**FIGURE 2:**
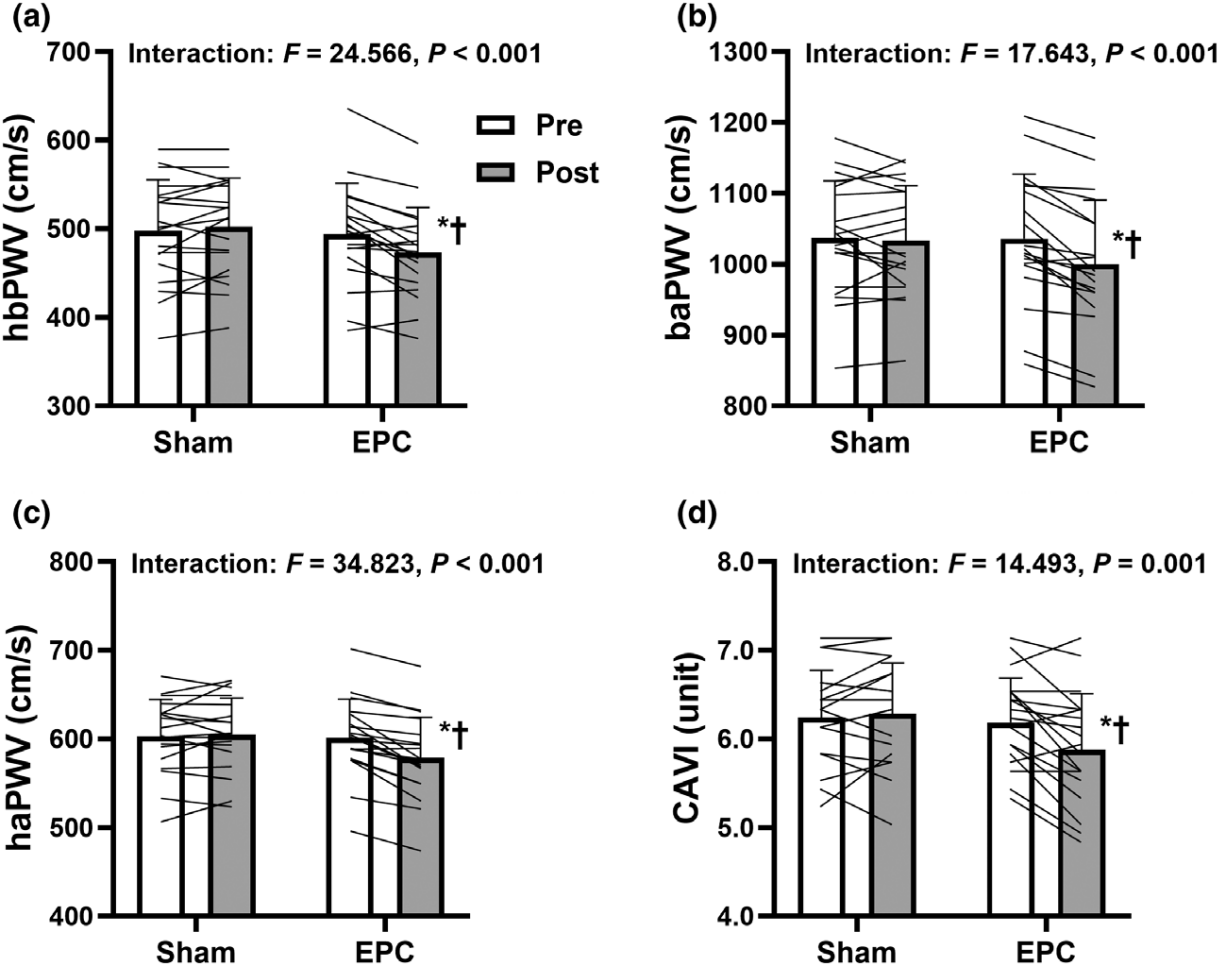
Measurements of hbPWV (a), baPWV (b), haPWV (c), and CAVI (d). baPWV, brachial‐ankle pulse wave velocity; CAVI, cardio‐ankle vascular index; EPC, external pneumatic compression; haPWV, heart‐ankle pulse wave velocity; hbPWV, heart‐brachial pulse wave velocity. *p < 0.05 versus pre. ^†^ *p* < 0.05 versus the same time‐point in the sham trial. Data are expressed as mean ± standard deviation.

Table 2 shows the hemodynamic response to treatment and none of the parameters exhibited a significant change over time for any trial. Table 3 presents the time‐course changes in sAA activity. The sAA level decreased significantly at T2 and at Post relative to Pre in the EPC trial, whereas no significant alteration was evident over time in the sham trial. At T2 and Post, sAA activity was significantly higher in the EPC trial than in the sham trial. Figure 2 shows the Pre–Post changes in arterial stiffness measures. In the EPC trial, all measures significantly decreased at Post relative to Pre (dz = 1.00, 1.37, 1.48, and 0.85 for hbPWV, baPWV, haPWV, and CAVI, respectively), but no such changes were observed in the sham trial (dz = 0.28, 0.14, 0.13, and 0.19 for hbPWV, baPWV, haPWV, and CAVI, respectively). At Post, all parameters exhibited a significantly lower value in the EPC trial compared to the sham trial. The Pre–Post changes in hemodynamic variables are presented in Table 4 and no significant change was found in any of these variables for both trials. Table 5 shows Pre–Post changes in mood. In both trials, tension and fatigue significantly decreased at Post relative to Pre, respectively. Anxiety and relaxation decreased significantly at Post relative to Pre only in the EPC trial. At Post, the level of relaxation was significantly higher in the EPC trial than in the sham trial.

**TABLE 2:** Hemodynamic response to the study treatment.

	Trial	Pre	T1	T2	ANOVA
Heart rate (bpm)	Sham	62 ± 8	62 ± 7	62 ± 7	Time: *F* = 1.247, *p* = 0.300; Trial: *F* = 0.002, *p* = 0.968
	EPC	63 ± 8	62 ± 6	62 ± 6	Interaction: *F* = 0.262, *p* = 0.771
Systolic blood pressure (mmHg)	Sham	108 ± 9	108 ± 9	108 ± 10	Time: *F* = 0.581, *p* = 0.565; Trial: *F* = 0.121, *p* = 0.733
	EPC	108 ± 8	109 ± 9	108 ± 8	Interaction: *F* = 0.247, *p* = 0.782
Diastolic blood pressure (mmHg)	Sham	63 ± 8	63 ± 7	62 ± 8	Time: *F* = 0.355, *p* = 0.704; Trial: *F* = 0.364, *p* = 0.554
	EPC	62 ± 8	61 ± 8	63 ± 8	Interaction: *F* = 2.319, *p* = 0.113
Mean arterial pressure (mmHg)	Sham	78 ± 8	78 ± 7	77 ± 8	Time: *F* = 0.182, *p* = 0.834; Trial: *F* = 0.133, *p* = 0.719
	EPC	77 ± 7	77 ± 7	78 ± 8	Interaction: *F* = 1.744, *p* = 0.189

*Note*: Data are expressed as mean ± standard deviation. Data were measured using the automated sphygmomanometer (i.e., the Tango device) in the seated position. T1 and T2, 10–12 and 20–22 min following treatment initiation, respectively.

Abbreviations: ANOVA, analysis of variance; EPC, external pneumatic compression.

**TABLE 3:** Time‐course changes in sAA activity.

	Trial	Pre	T1	T2	Post	ANOVA
sAA activity (kU/L)	Sham	19 ± 12	19 ± 13	19 ± 11	19 ± 12	Time: *F* = 4.313, *p* = 0.008; Trial: *F* = 5.783, *p* = 0.027
	EPC	20 ± 14	18 ± 13	13 ± 10^a^, ^b^	12 ± 8^a^, ^b^	Interaction: *F* = 6.369, *p* < 0.001

*Note*: Data are expressed as mean ± standard deviation. T1 and T2, 10–12 and 20–22 min following treatment initiation, respectively.

Abbreviations: ANOVA, analysis of variance; EPC, external pneumatic compression; sAA, salivary alpha‐amylase.

^a^ *p* < 0.05 versus pre.

^b^ *p* < 0.05 versus the same time‐point in the sham trial.

**TABLE 4:** Pre–post changes in hemodynamic variables.

	Trial	Pre	Post	ANOVA
Heart rate (bpm)	Sham	59 ± 7	59 ± 6	Time: *F* = 0.005, *p* = 0.943; Trial: *F* = 1.931, *p* = 0.182
	EPC	60 ± 7	60 ± 7	Interaction: *F* = 1.456, *p* = 0.243
Systolic blood pressure (mmHg)	Sham	112 ± 11	113 ± 12	Time: *F* = 0.216, *p* = 0.648; Trial: *F* = 0.164, *p* = 0.690
	EPC	113 ± 10	111 ± 10	Interaction: *F* = 3.462, *p* = 0.079
Diastolic blood pressure (mmHg)	Sham	69 ± 6	69 ± 6	Time: *F* = 0.430, *p* = 0.520; Trial: *F* = 1.104, *p* = 0.307
	EPC	68 ± 7	68 ± 7	Interaction: *F* = 0.064, *p* = 0.803
Mean arterial pressure (mmHg)	Sham	84 ± 7	84 ± 7	Time: *F* = 0.096, *p* = 0.760; Trial: *F* = 1.400, *p* = 0.252
	EPC	84 ± 6	83 ± 7	Interaction: *F* = 1.710, *p* = 0.208

*Note*: Data are expressed as mean ± standard deviation. Data were measured using the vascular screening system (i.e., the VaSera system) in the supine position.

Abbreviations: ANOVA, analysis of variance; EPC, external pneumatic compression.

**TABLE 5:** Mood outcomes.

	Trial	Pre	Post	ANOVA
Tension	Sham	3.1 ± 1.3	2.5 ± 0.9^a^	Time: *F* = 30.516, *p* < 0.001; Trial: *F* = 0.033, *p* = 0.858
	EPC	3.3 ± 1.0	2.4 ± 0.8^a^	Interaction: *F* = 2.070, *p* = 0.167
Anxiety	Sham	3.1 ± 1.1	2.8 ± 1.2	Time: *F* = 20.535, *p* < 0.001; Trial: *F* = 0.095, *p* = 0.761
	EPC	3.2 ± 1.1	2.5 ± 0.8^a^	Interaction: *F* = 4.800, *p* = 0.042
Fatigue	Sham	4.5 ± 1.4	4.1 ± 0.7^a^	Time: *F* = 7.691, *p* = 0.013; Trial: *F* = 1.884, *p* = 0.187
	EPC	4.4 ± 1.5	3.7 ± 1.2^a^	Interaction: *F* = 0.597, *p* = 0.450
Relaxation	Sham	6.4 ± 0.9	6.2 ± 0.9	Time: *F* = 3.894, *p* = 0.064; Trial: *F* = 4.679, *p* = 0.044
	EPC	6.2 ± 0.9	7.1 ± 0.8^a^, ^b^	Interaction: *F* = 15.190, *p* = 0.001

*Note*: Data are expressed as mean ± standard deviation.

Abbreviations: ANOVA, analysis of variance; EPC, external pneumatic compression.

^a^ *p* < 0.05 versus pre.

^b^ *p* < 0.05 versus the same time‐point in the sham trial.

## 4 DISCUSSION

The most salient finding of this study is the observation of a significant reduction in hbPWV, baPWV, haPWV, and CAVI after 30 min of EPC treatment, indicating that EPC decreased arterial stiffness not only in the segments covering the treated sites but also in the untreated segment. Our findings suggest that in healthy young adults, a single session of lower‐limb peristaltic pulse EPC can acutely ameliorate arterial stiffness, which would occur at a systemic level.

In the present study, baPWV, which reflects the stiffness of the abdominal aorta and leg arteries (Stone et al., 2023; Sugawara et al., 2018), significantly decreased following the 30‐min EPC treatment. Since acute changes in arterial stiffness could be driven by changes in endothelial function (Stoner et al., 2020), the present finding is mechanistically supported by previous studies that found improvements in endothelial function in the popliteal artery, as evidenced by an increase in FMD response, after lower‐limb EPC lasting 30 or 60 min (Martin et al., 2015, 2018). Studies have suggested that the main factor inducing improvement in FMD within the treated limb may be changes in hemodynamic shear stress and consequently, local nitric oxide (NO) bioavailability during EPC treatment (Martin et al., 2018). Indeed, the mRNA and protein expression levels of endothelial NO synthase and NO metabolite accumulation tended to be augmented in the vastus lateralis biopsy samples collected following a 60‐min bout of lower‐limb EPC (Kephart et al., 2015). However, no alterations in heart rate were observed during the current EPC protocol (Table 1). In this regard, although different from lower‐limb compression, passive static stretching has similarities to EPC; specifically, both are passive techniques involving alternating compressed/stretching and deflated/relaxation phases. In the case of passive static stretching of the calf, blood flow in the posterior tibial artery increased temporally after stretching for 30 s, whereas changes in heart rate and cardiac output were not observed during both the stretching and subsequent relaxation phases (Higaki et al., 2021). Therefore, we consider that without changes in central circulation, EPC increased hemodynamic shear stress in the lower limb during the cycle of deflation (e.g., mild‐to‐moderate reactive hyperemia) (Martin et al., 2015), resulting in the reduction in arterial stiffness in the same sites via the NO mechanism. This potential vascular alteration seems likely to be a contributing factor to the decrease in baPWV observed in the present study. This also applies to the present result of a decrease in systemic indices of arterial stiffness (i.e., haPWV and CAVI) caused by the EPC session, as these include information on the leg artery segment (Hayashi et al., 2015).

We further found a significant decrease in hbPWV after 30‐min EPC treatment. The EPC‐induced enhancements in FMD in untreated limbs have been reported previously (Martin et al., 2015, 2018). Similarly, a 30‐min massage therapy of the lower limb has been shown to increase brachial artery FMD (Franklin et al., 2014). Thus, a reduction in arterial stiffness in the upper extremity may have contributed to the current observation with respect to hbPWV. From another perspective, the projected arterial segment evaluated by hbPWV covers not only muscular arteries in the upper extremity but also the proximal aorta (Sugawara et al., 2018, 2019), and consequently, hbPWV can serve as a marker of proximal aortic stiffness (Sugawara et al., 2018, 2019). Therefore, the observed decrease in hbPWV supposably implies destiffening of the proximal aortic region. However, the question regarding the mechanism(s) underpinning the amelioration of arterial stiffness in untreated sites remains. It has been reported that leg exercise on a recumbent cycle ergometer did not significantly increase blood flow in the brachial artery, even when the heart rate was elevated to approximately 100 bpm (Tanaka et al., 2006). Although brachial artery blood flow was not measured in this study, we suspect that significant hemodynamic changes in the upper extremity did not occur during the current EPC session, similar to the central circulation. Thus, unlike the other arterial stiffness parameters assessed in the present study, the increase in hemodynamic shear stress was probably not the main cause of the decrease in hbPWV.

Another possibility is the alteration in sympathetic nerve activity, which can modulate arterial stiffness via changes in vascular smooth muscle tone (Nardone et al., 2020). In our study, sAA activity, measured as a surrogate marker of sympathetic nerve activity, decreased gradually over time during the 30‐min EPC session, while a significant reduction was noted at T2 and Post. Our finding is supported by one previous study, which indicated reduced sAA levels after a single session of traditional Thai massage (Sripongngam et al., 2015) and another study that demonstrated a soothing video viewing‐induced decrease in sAA levels (Takai et al., 2004). Positive changes in mood have been suggested to be associated with a decrease in sympathetic nerve activity (Kim & Song, 2022; Song et al., 2017). In the present study, EPC treatment was found to be more effective in immediately improving mood than the sham treatment in some assessment items. This is in line with the findings in the case of leg massage therapy (Noto et al., 2010). Collectively, although largely speculative, the decrease in hbPWV induced by EPC was probably due to reduced sympathetic activation via beneficial mood alterations. Our speculation may be supported by a study showing that mirthful laughter induced by the viewing of comedy movies led to a temporal improvement in arterial stiffness (Vlachopoulos et al., 2009). Considering that sympathetic modulation acts systemically (Imadojemu et al., 2001; Rea & Wallin, 1989), its effect might partially be involved in the decrease in baPWV, haPWV, and CAVI after the EPC session as well.

Acute passive stretching protocols reportedly ameliorate arterial stiffness only in the stretched sites without improving the non‐stretched sites (Ikebe et al., 2022; Yamato et al., 2017). Moreover, electrical muscle stimulation of the lower extremities has been indicated to induce a reduction in arterial stiffness only in sites that received stimulation (Oda et al., 2022). In contrast with these prior studies, in the present study, the EPC session decreased arterial stiffness in the untreated segment, in addition to the segments covering the treated sites. Although this potential systemic vascular effect may be obtained from lower‐limb massage (Franklin et al., 2014), EPC devices have the advantage of not requiring manpower, making them easier to implement in daily life.

The present study demonstrated an acute reduction in arterial stiffness induced by a single session of lower‐limb peristaltic pulse EPC; however, its chronic vascular effects remain unclear. The changes in FMD in response to acute exercise were associated with changes in resting FMD following exercise training (Dawson et al., 2018), indicating that acute vascular responses to physiological stimuli could be related to chronic vascular adaptation to their repetition. Moreover, although the degree of pressure and compression modalities is different from the EPC device used in this study, chronic administration of enhanced external counterpulsation has been reported to elicit a significant reduction in arterial stiffness in clinical patients (Casey et al., 2011). Taken together, it is possible that habitual EPC treatment induces amelioration of the basal levels of arterial stiffness. Therefore, longitudinal EPC intervention studies are needed to clarify the chronic vascular effects and their clinical significance. The study findings will be helpful in establishing simple and effective passive approaches for reducing arterial stiffness.

### 4.1 Limitations

The present study has some limitations. First, only young healthy individuals were included in this study. Therefore, future studies should examine whether the present findings can be generalized to other populations such as middle‐aged and older adults and patients with various conditions. Second, we did not control for the menstrual cycle of female participants, which could affect the present results (Okamoto et al., 2017). Third, we performed sAA measurements as a surrogate assessment of sympathetic nerve activity, but it has been recently suggested that sAA could broadly represent the activity of the autonomic nerve system rather than sympathetic nerve activity alone (Ali & Nater, 2020). Therefore, caution should be exercised in interpreting the present results of sAA levels, and it should ideally be evaluated using more robust markers, such as muscle sympathetic nerve activity or plasma norepinephrine levels (Grassi & Esler, 1999), in future studies. Fourth, a lack of blood flow or shear rate data for the upper and lower limbs during EPC limits our ability to draw definitive conclusions in this aspect. To address this limitation and elucidate the physiological mechanisms of the EPC‐induced arterial stiffness reduction, future studies should integrate arterial stiffness assessments with regional hemodynamic evaluations using ultrasonography.

## 5 CONCLUSION

The present study revealed a significant decrease in hbPWV, baPWV, haPWV, and CAVI induced by a 30‐min EPC session. These findings suggest that in healthy young adults, an acute bout of lower‐limb peristaltic pulse EPC can decrease arterial stiffness, which would be elicited systemically.

## AUTHOR CONTRIBUTIONS

DK conceived and designed the study, performed the experiments, and analyzed the data. DK, MN, and TM interpreted the results of the experiments and drafted the manuscript. DK and MN edited and revised the manuscript. All authors approved the final version of the manuscript.

## FUNDING INFORMATION

This work was supported by JSPS KAKENHI Grant Number 23K10649 (to DK).

## CONFLICT OF INTEREST STATEMENT

The authors have no conflict of interest to declare.

## Data Availability

The datasets generated and analyzed during the current study are available from the corresponding authors upon reasonable request.
